# Isopropanol biosynthesis from crude glycerol using fatty acid precursors via engineered oleaginous yeast *Yarrowia lipolytica*

**DOI:** 10.1186/s12934-022-01890-6

**Published:** 2022-08-19

**Authors:** Xiaoyu Shi, Hyeon Min Park, Minhye Kim, Myeong-Eun Lee, Wu-Young Jeong, Joonhee Chang, Byeong-Hyeon Cho, Sung Ok Han

**Affiliations:** Department of Biotechnology, Korea University, Seoul, 02841 Republic of Korea

**Keywords:** Crude glycerol, Isopropanol, *Yarrowia lipolytica*, Metabolic engineering, Malonyl-CoA

## Abstract

**Background:**

Isopropanol is widely used as a biofuel and a disinfectant. Chemical preparation of isopropanol destroys the environment, which makes biological preparation of isopropanol necessary. Previous studies focused on the use of expensive glucose as raw material. Therefore, the microbial cell factory that ferments isopropanol with cheap raw materials will provide a greener way to produce isopropanol.

**Results:**

This study converted crude glycerol into isopropanol using *Y. lipolytica*. As a microbial factory, the active natural lipid and fatty acid synthesis pathway endows *Y. lipolytica* with high malonyl-CoA production capacity. Acetoacetyl-CoA synthase (*nphT7*) and isopropanol synthesis genes are integrated into the *Y. lipolytica* genome. The *nphT7* gene uses the accumulated malonyl-CoA to synthesize acetoacetyl-CoA, which increases isopropanol production. After medium optimization, the best glycerol medium was found and resulted in a 4.47-fold increase in isopropanol production. Fermenter cultivation with pure glycerol medium resulted in a maximum isopropanol production of 1.94 g/L. In a crude glycerol fermenter, 1.60 g/L isopropanol was obtained, 82.53% of that achieved with pure glycerol. The engineered *Y. lipolytica* in this study has the highest isopropanol titer reported.

**Conclusions:**

The engineered *Y. lipolytica* successfully produced isopropanol by using crude glycerol as a cheap carbon source. This is the first study demonstrating the use of *Y. lipolytica* as a cell factory to produce isopropanol. In addition, this is also a new attempt to accumulate lipid synthesis precursors to synthesize other useful chemicals by integrating exogenous genes in *Y. lipolytica*.

**Supplementary Information:**

The online version contains supplementary material available at 10.1186/s12934-022-01890-6.

## Background

The production of valuable biofuels and biochemicals from industrial byproducts has become a social concern [1]. Isopropanol is industrially converted to propylene, which is important as a biofuel. The current production of isopropanol mainly occurs through two main processes, chemical industrial production [2, 3] and biosynthesis [4, 5]. Chemical methods pollute the environment, which makes the biosynthesis of isopropanol a more attractive option.

In *Escherichia coli*, 4.9 g/L of isopropanol was produced by expressing various combinations of genes, such as *thl* (acetyl-coenzyme A [CoA] acetyltransferase), *atoDA* (acetoacetyl-CoA transferase), *adc* (acetoacetate decarboxylase), and *adh* (secondary alcohol dehydrogenase) [6]. It has also been reported that the yeast strain *Candida utilis* produced 1.2 g/L isopropanol [7]. Since the strains mentioned above take a long time and use expensive glucose as a feedstock, a more efficient and cost-effective feedstock for isopropanol biosynthesis is desirable.

In cyanobacteria, isopropanol was produced from acetate, but due to stringent requirements for anaerobic growth conditions, only 0.146 g/L isopropanol was produced in 10 days [8]. Yang et al. reported 1.47 g/L isopropanol production using acetate as a carbon source in *E. coli* [9], which is not ideal compared to isopropanol production utilizing glucose. Therefore, it is a challenge to select a less valuable carbon source among the various carbon sources for isopropanol production that can tolerate toxic alcohol culture conditions as well.

**Fig. 1:**
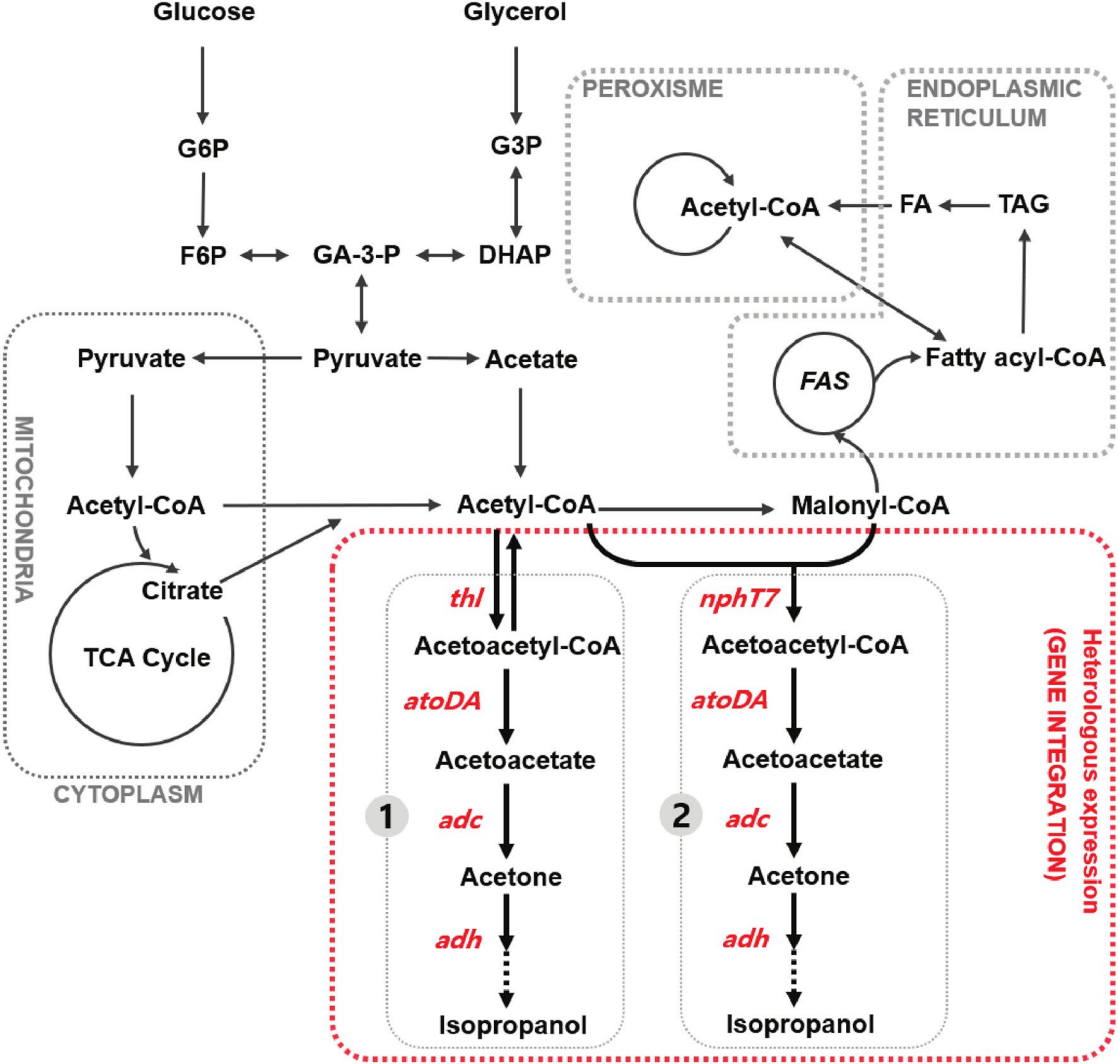
Metabolic engineering strategy for isopropanol production in *Y. lipolytica.* The abbreviations correspond to acetyl-CoA acetyltransferase (*thl*), acetoacetyl-CoA synthase (*nphT7*), acetoacetyl-CoA transferase (*atoDA*), acetoacetate decarboxylase (*adc*), and secondary alcohol dehydrogenase (*adh*)

When combined with the biofuel production chain, crude glycerol generated during biodiesel, was chosen as a cheap feedstock for isopropanol production. *Y. lipolytica*, known as a GRAS (generally recognized as safe) status strain, was selected, since its genome can be manipulated by molecular biological technique [10]. Most importantly, it grows well on various carbon sources, especially glycerol and its tolerance to environmental factors such as unstable pH and various alcohol toxicities makes it an appropriate choice [11, 12]. The *thl* gene uses two acetyl-CoA to synthesize an important precursor in the isopropanol synthetic pathway, acetoacetyl-CoA. The amount of synthesized acetoacetyl-CoA is critical to increasing the isopropanol yield [13]. The *nphT7* gene has been reported to synthesize acetoacetyl-CoA from malonyl-CoA and acetyl-CoA. Additionally, *nphT7* can be used to significantly increase acetoacetyl-CoA concentrations in *E. coli* [14]. Malonyl-CoA and acetyl-CoA are important substrates for *Y. lipolytica* in the synthesis of lipids and fatty acids [15]. Because of the relatively high natural acetyl-CoA level, engineered *Y. lipolytica* can be used to biosynthesize isopropanol with high efficiency using cheap crude glycerol waste by rationally utilizing its lipid synthesis pathway (Fig. 1). This was first reported for the synthesis of isopropanol from waste crude glycerol, as well as for the initial synthesis of isopropanol using the lipid synthesis pathway in *Y. lipolytica*.

## Results and discussion

### Heterologous overexpression of acetoacetyl-CoA synthase improves isopropanol production

**Fig. 2:**
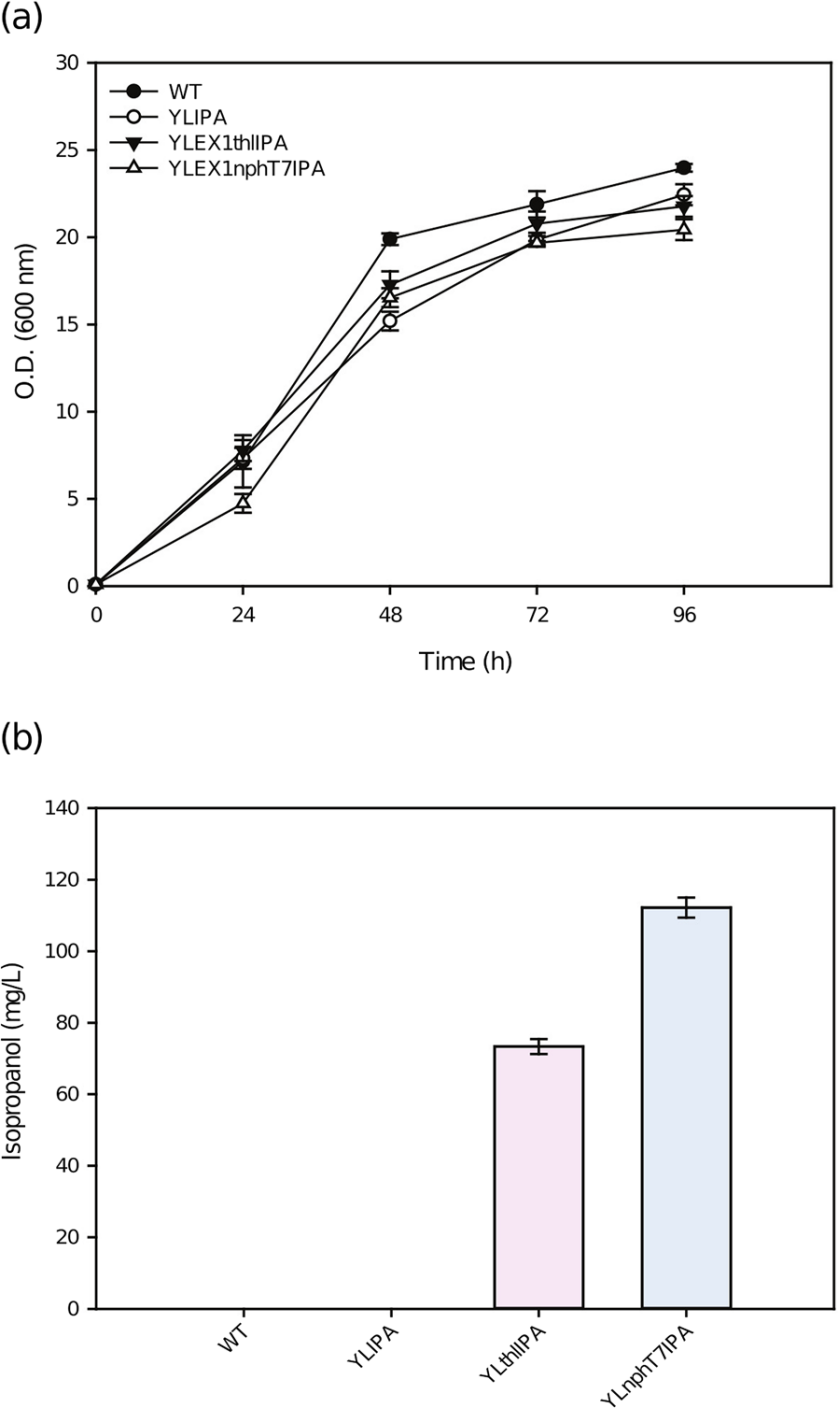
Enhanced isopropanol production by heterologous overexpression acetoacetyl-CoA synthase*.* Wild-type *Y. lipolytica.* strains and engineered *Y. lipolytica.* strains YLIPA, YLthlIPA, and YLnphT7IPA were cultivated in 250 ml baffled flasks in YPG20 medium at 30 °C and 200 rpm after 96 h. **a** Growth curves, **b** isopropanol production of the strains

As shown in Fig. 2a, by inserting the traditional isopropanol synthetic pathway into *Y. lipolytica,* the engineered strain YLthlIPA was developed, and this strain produced 73.35 mg/L isopropanol. The wild-type strain and YLIPA strain, which did not overexpress the *thl* gene, did not produce isopropanol.

Because of the suboptimal isopropanol yield in *Y. lipolytica*, adaptation at the genetic engineering level is required to overcome such limitations. As an oleaginous yeast, *Y. lipolytica* has excellent malonyl-CoA production ability [16]. Therefore, *nphT7* gene was used instead of the *thl* gene to increase acetoacetyl-CoA synthesis. The *nphT7* gene uses malonyl-CoA in *Y. lipolytica* and reasonably produces isopropanol, which can solve the problems at the gene level and improve the yield of isopropanol.

As shown in Fig. 2a, comparing the YLthlIPA strain with the YLnphT7IPA strain, their growth curves were not significantly different, but as shown in Fig. 2b, after heterologous expression of the *nphT7* gene, the engineered strain YLnphT7IPA produced 112 mg/L isopropanol, which was 1.53-fold higher than the YLthlIPA strain. Isopropanol production was greatly increased in the YLnphT7IPA strain. This illustrates that the single cell isopropanol production capacity of YLnphT7IPA strain is stronger than that of YLthlIPA strain. This also confirms that heterologous expression of the *nphT7* gene in *Y. lipolytica* was a correct attempt (Table 1).

**Table 1:** Isopropanol yields to determine the optimal medium for Box–Behnken

Run	Yeast extract (g/L)	Peptone (g/L)	Glycerol (g/L)	Isopropanol (mg/L)
1	20.00	35.00	70.00	482.000
2	30.00	35.00	120.00	164.500
3	20.00	35.00	70.00	313.500
4	10.00	35.00	120.00	113.120
5	20.00	35.00	70.00	460.100
6	30.00	10.00	70.00	96.354
7	20.00	35.00	70.00	455.300
8	20.00	60.00	20.00	43.120
9	10.00	35.00	20.00	62.220
10	20.00	10.00	120.00	85.582
11	20.00	60.00	120.00	97.790
12	10.00	60.00	132.50	122.460
13	20.00	10.00	70.00	39.330
14	30.00	35.00	20.00	66.880
15	10.00	10.00	70.00	251.900
16	20.00	35.00	70.00	452.100
17	30.00	60.00	70.00	310.000

### Response surface methodology (RSM) for medium optimization increased isopropanol yield

To further increase isopropanol production, medium optimization was conducted using the most productive YLnphT7IPA strain. YPD medium is the most commonly used medium in yeasts, especially for culturing yeasts in large-scale industrial production [17]. When the YPD medium was optimized, the effect of the concentration of individual components on the yield of the product was significant, and the optimized medium improved the yield of the target product to some extent [18]. To make the YLnphT7IPA strain consume glycerol to produce isopropanol in large quantities, YPG medium was selected. Glycerol was used as a carbon source instead of glucose in the YPD medium. For the YPG medium, it is important to determine the optimal concentration of glycerol and coordinate the concentrations of the other components, such as peptone and yeast extract. To explore the interrelationship of the three factors, the RSM was selected for medium optimization. The reported studies on optimization of glycerol medium by the RSM method mostly investigated improvement of the yield of lipids [19].

The influencing factors of the culture medium were determined by Box–Behnken RSM (Table 2), and the experimental results were analyzed by analysis of variance. According to the calculation of the final equation, the optimal concentrations of various components in the culture medium for the maximum yield of isopropanol were obtained. A fitted equation in terms of the coded values for the prediction of isopropanol production was obtained. A, B, and C represent yeast extract, peptone, and glycerol, respectively, and can be expressed as follows in Eq. (1):

1 $${\text{Isopropanol}}\;({\text{mg}}/{\text{L}}) = 432.60 + 11.00*{\text{A}} + 12.53*{\text{B}} + 31.18*{\text{C}} + 85.77*{\text{A}}*{\text{B}} + 11.68*{\text{A}}*{\text{C}} + 2.10*{\text{B}}*{\text{C}} - 101.10*{\text{A}}^{2} - 136.32*{\text{B}}^{2} - 229.82*{\text{C}}^{2}$$

**Table 2:** Level of factors chosen for the central composite design response surface methodology and ANOVA of the model obtained for the optimal medium

Factor	Name	Low actual	High actual	Low coded	High coded	Mean	Std. dev
A	Yeast extract (g/L)	10.00	30.00	− 1.000	1.000	20.00	6.880
B	Peptone (g/L)	10.00	60.00	− 1.000	1.000	35.00	17.150
C	Glycerol (g/L)	20.00	120.00	− 1.000	1.000	70.00	34.300

Source	Sum of squares	Df	Mean square	F value	P value Prob > F	
Model	4.179E + 005	9	46,427.88	16.91	0.0006	Significant
A-Yeast extract	968.68	1	968.68	0.35	0.6141	
B-Peptone	1255.11	1	1255.11	0.46	0.5206	
C-Glycerol	7777.85	1	7777.85	2.83	0.1362	
AB	29,427.00	1	29,427.00	10.72	0.0136	
AC	6171E-003	1	6.171E-003	0.017	0.8994	
BC	17.72	1	17.72	6.454E-003	0.9382	
A^2^	43,035.08	1	3035.08	15.68	0.0055	
B^2^	78,248.68	1	78,248.68	28.51	0.0011	
C^2^	2.224E + 005	1	2.224E + 005	81.02	< 0.0001	
Lack of Fit	937.03	3	312.34	0.068	0.735	Not significant
R^2^				0.9560		
Adj R^2^				0.8995		
Pred R^2^				0.9004		
*Adeq. Precision*				10.147		

**Fig. 3:**
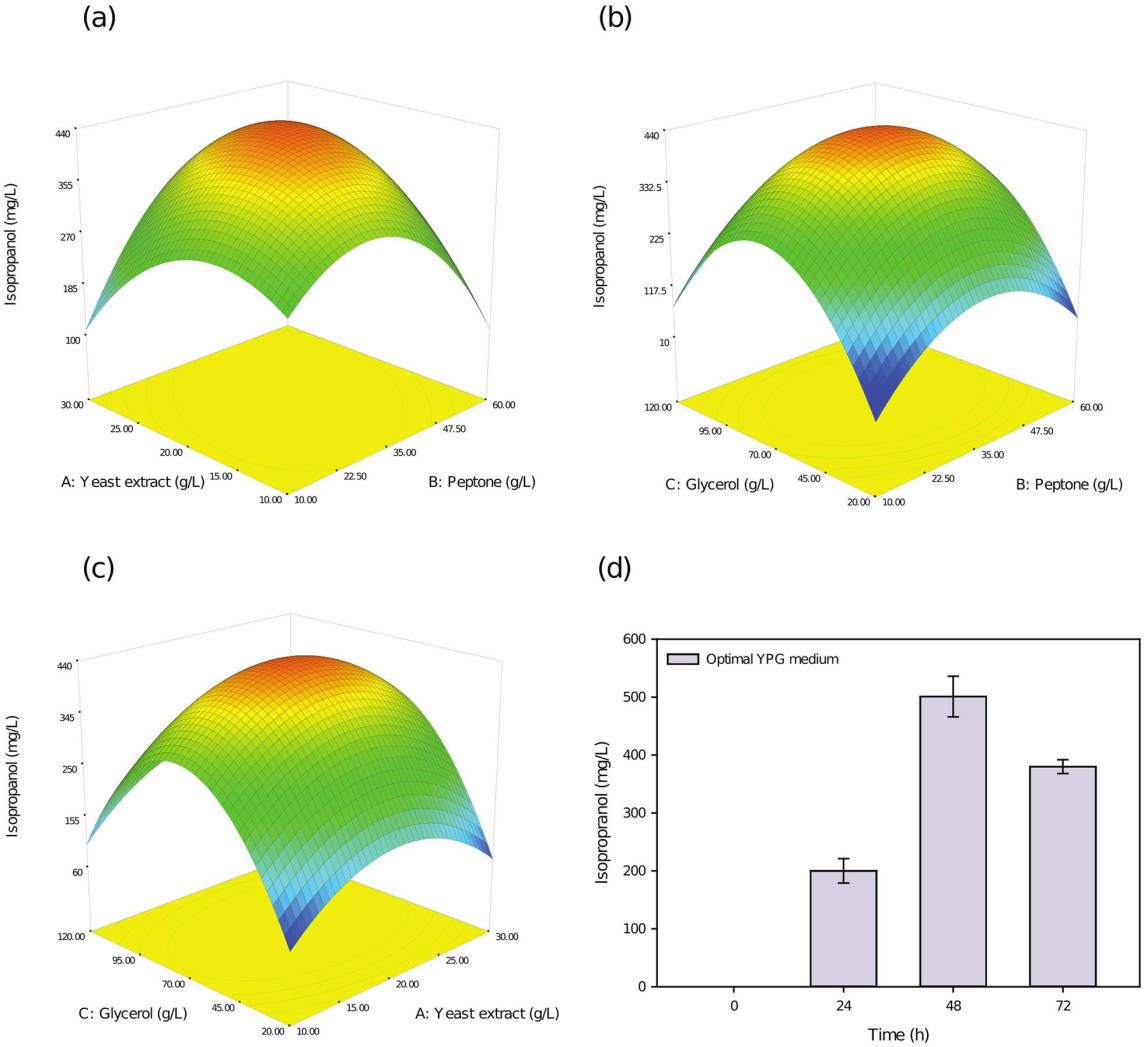
Glycerol medium optimization by response surface methodology to increase isopropanol production. **a** Effects of yeast extract and peptone, **b** effects of glycerol and peptone, and **c** effects of glycerol and yeast extract. **d** Cultivated YLnphT7IPA strain in 250 ml baffled flasks in optimal YPG medium at 30 °C and 200 rpm after 72 h. Optical density was measured at 600 nm every 24 h and was conducted in triplicate

The 3D surface diagrams of the two factors are shown in Fig. 3a–c. The optimal medium concentrations were 20.90 g/L yeast extract, 36.85 g/L peptone and 73.53 g/L glycerol. The maximum isopropanol production was 482 mg/L. The predicted medium concentration was used to verify the experiment. As shown in Fig. 3d, the actual isopropanol yield was 501.00 ± 35.00 mg/L after 48 h, which was in good agreement with the prediction and verifies the effectiveness of the model. However, as shown in Fig. 2b, 112 mg/L isopropanol was produced in the nonoptimized medium. Medium optimization resulted in 4.47-fold increased isopropanol production. The YPG medium before and after optimization contained 20 g/L and 70 g/L glycerol, respectively. Glycerol was added to the medium at the optimum concentration, which increased the C/N ratio of the medium. There is no research report on the medium optimization method for the large-scale production of isopropanol with glycerol. However it has been reported that increasing the C/N ratio of the culture medium contributes to the supply of acetyl-CoA in *Y. lipolytica* [20]. The increase in the synthesis amount of acetyl-CoA is helpful to improve the yield of isopropanol. Therefore, it concludes that increasing the C/N ratio in the medium will improve the isopropanol yield.

### Batch cultivation in 5 L fermenter using pure glycerol

**Fig. 4:**
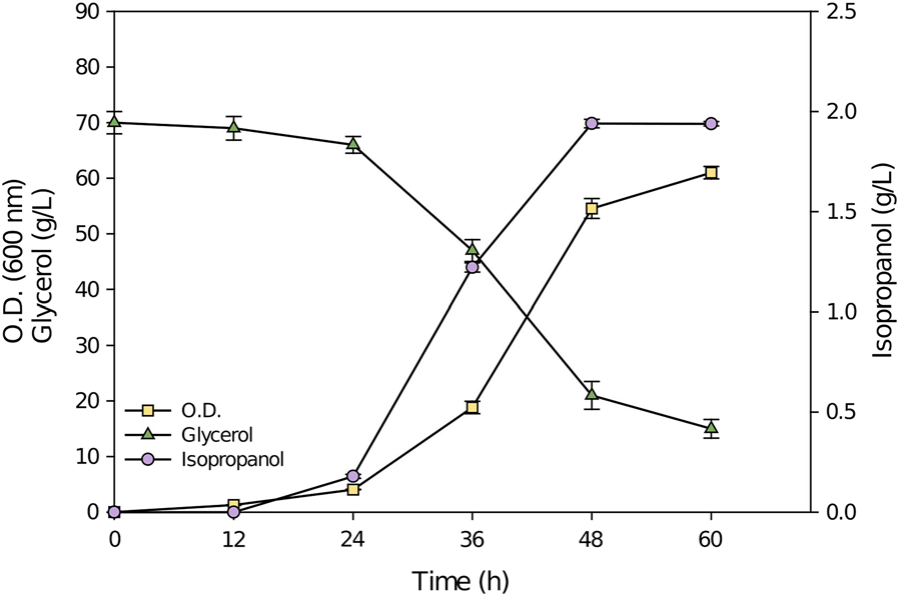
The YLnphT7IPA strain was cultured in a 5 L fermenter using optimal pure glycerol medium. The quantification was conducted in triplicate. Lines in yellow, purple, and green represent the growth curves, isopropanol production and glycerol consumption, respectively

Optimized medium was used to culture the engineered YLnphT7IPA strain in a 5 L fermenter. Figure 4 shows that after 48 h of culture, the yield of isopropanol reached a maximum of 1.94 g/L. Carefully observing the growth of cells, it was found that although the production of isopropanol was highest at 48 h, the number of cells was not the highest. It was found that in the first 48 h, *Y. lipolytica* mainly consumes glycerol for cell growth and for isopropanol synthesis. After 48 h, the glycerol consumed by *Y. lipolytica* was mainly used to produce lipids, and the production of isopropanol began to decrease accordingly. It has been reported that when cultured in a fermenter using glycerol as a carbon source, the cells begin to produce lipids and fatty acids at 48 h [21, 22]. This is also in line with our expectation that the heterologous expression of the *nphT7* gene utilizes the precursors of lipid synthesis, acetyl-CoA and malonyl-CoA.

### YLnphT7 strain cultured in glucose medium

**Fig. 5:**
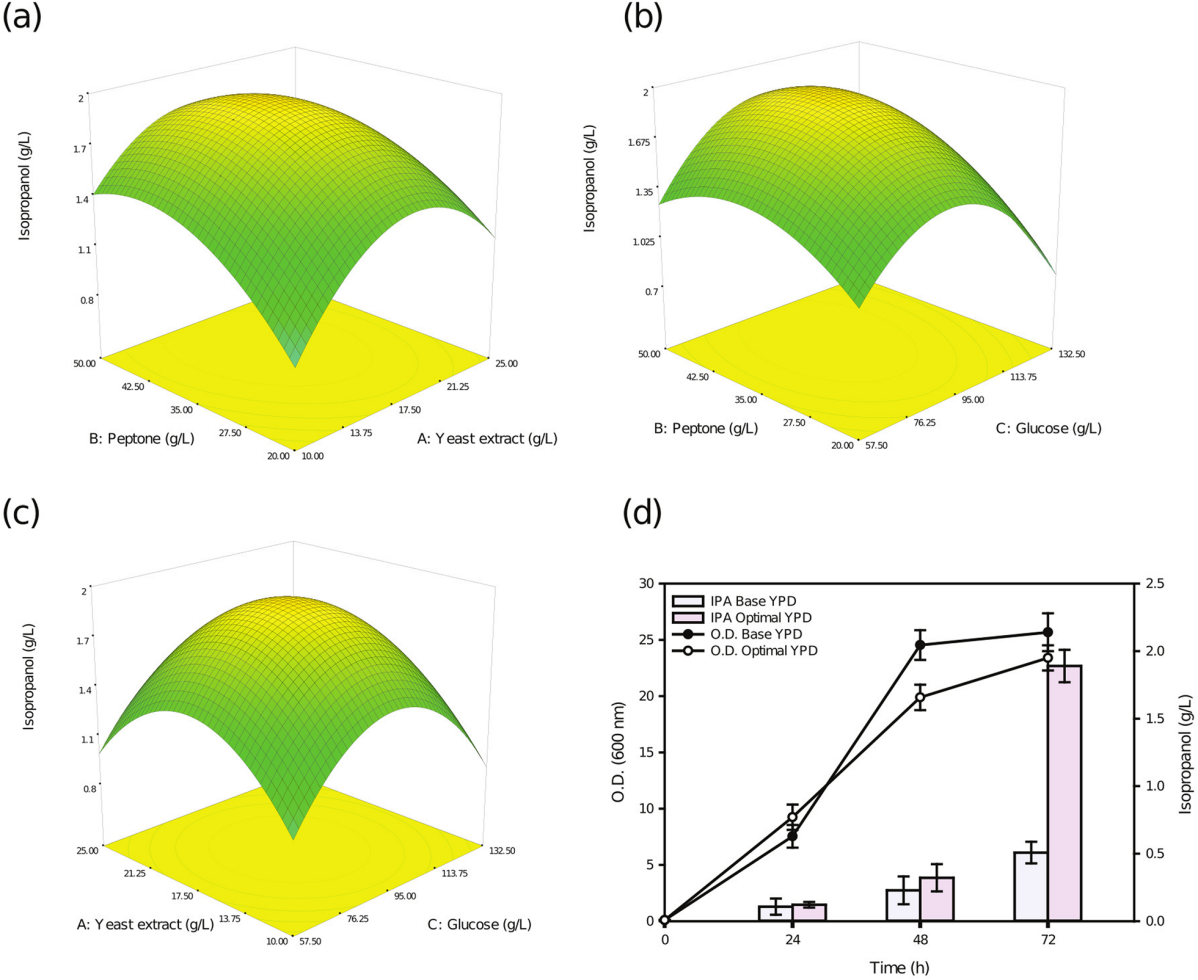
The YLnphT7 strain was cultured in optimal glucose medium. **a** Effects of yeast extract and peptone, **b** effects of glucose and peptone, and **c** effects of glucose and yeast extract. **d** Cultivated YLnphT7IPA strain in 250 ml baffled flasks in optimal YPD medium at 30 °C and 200 rpm after 72 h. The quantification was conducted in triplicate

For comparison, YLnphT7IPA strain was cultured in medium using glucose as a carbon source. The medium was optimized by RSM (Additional file 1: Table S4) and cultured with the optimized glucose medium in a 250 ml flask (Fig. 5a–c). After 72 h of culture, a maximum of 1.89 g/L isopropanol was obtained (Fig. 5d). Then, the optimized medium was used for culture in the fermenter, and the maximum isopropanol yield of 821 mg/L was obtained in 24 h. After 24 h, isopropanol was not produced, although the cell concentration continued to increase (Additional file 1: Fig. S1).

According to previous reports, glycerol consumption and metabolism were preferentially observed when *Y. lipolytica* was cultured in a fermenter using a medium with a mixed carbon source of glucose and glycerol, and after 12 h of culture with glycerol, the culture reached oxygen limitation [23]. The preferential consumption of glycerol explains why the time of maximum isopropanol production is delayed when cultured in glucose medium (Fig. 5d). Oxygen limitation in glycerol medium is one of the obstacles to the synthesis of fatty acids and lipids by *Y. lipolytica* [24]. Oxygen restriction inhibits the latter half of the synthesis pathway of lipids and fatty acids. It is speculated that this restriction allows to synthesize more precursors of lipid and fatty acid, acetyl-CoA and malonyl-CoA, to be used in the synthesis of isopropanol. This is supposed that is why the medium with glycerol as a carbon source resulted in higher isopropanol production.

### Isopropanol was produced using crude glycerol as a carbon source

**Fig. 6:**
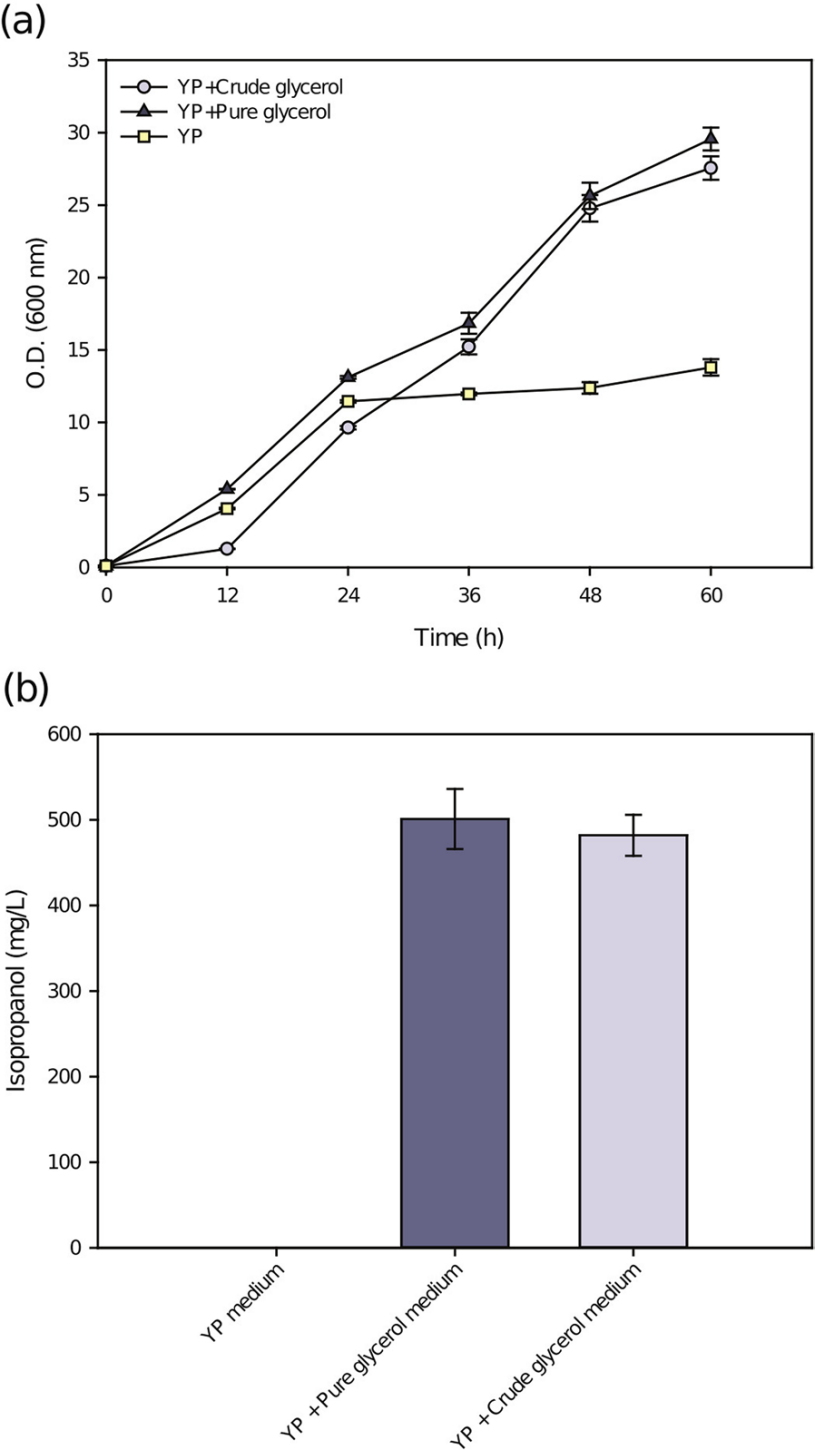
Fermentation of the YLnphT7IPA strain using optimal crude glycerol medium. **a** Growth curves, **b** isopropanol production and crude glycerol consumption of the YLnphT7IPA strain cultured in 250 ml flasks in 50 ml medium with YP medium, YP pure glycerol medium and YP-crude glycerol medium

Crude glycerol was added to the culture medium as a carbon source to test the isopropanol production capacity of the engineered strain YLnphT7IPA. As shown in Fig. 5, in the first 24 h, the strain mainly adapted to the growth environment, which was due to the influence of impurities in crude glycerol but the strain returned to the normal growth rate after 24 h (Fig. 6a). This is also consistent with a previous report on the growth adaptation of the *Y. lipolytica* strain to crude glycerol [25]. The maximum yield of 470 mg/L isopropanol was obtained after the strain was cultured in medium with crude glycerol as a carbon source for 48 h. However, as in the control group, the production of isopropanol was not observed in the basic YP medium without crude glycerol.

The consumption of glycerol in crude glycerol medium proves that the YLnphT7IPA strain can effectively use crude glycerol as a carbon source. Moreover, compared with the pure glycerol medium, the isopropanol yield of crude glycerol medium decreased by only 4.8% (Fig. 6b). The YLnphT7IPA strain obtained a similar yield to that of pure glycerol medium when crude glycerol was used as the carbon source. This proves the engineered strain could efficiently produce isopropanol from crude glycerol.

Previous reports indicate that refining crude glycerol into pure glycerol, whether chemically or biologically, requires a great deal of money and time [26]. Using crude glycerol as a carbon source, YLnphT7IPA can obtain an isopropanol yield similar to that in pure glycerol culture, indicating that a similar production capacity can be obtained without purifying crude glycerol to glycerol. The time required to reach the maximum isopropanol titer using crude glycerol medium was also similar to that when pure glycerol was used as the carbon source. The incubation time required for crude glycerol was shorter than that required for glucose.

### Batch cultivation in a 5 L fermenter using crude glycerol

**Fig. 7:**
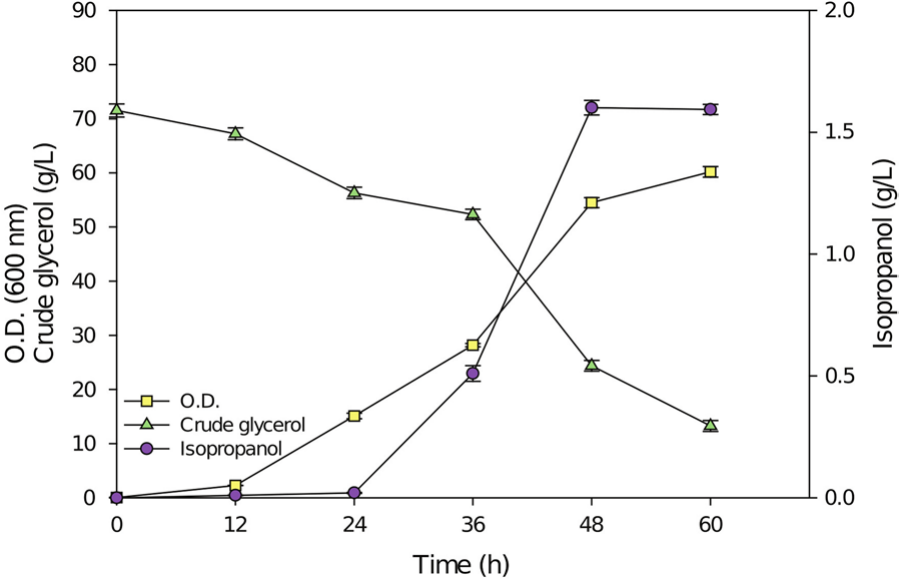
The YLnphT7IPA strain was cultured in a 5 L fermenter using crude glycerol. The YLnphT7IPA strain was cultured in a 5 L fermenter using crude glycerol as a carbon source. Lines in yellow, purple, and green represent the growth curves, isopropanol production and crude glycerol consumption, respectively. The quantification was conducted in triplicate

As mentioned earlier, crude glycerol causes the growth rate to slow down in the preculture period of the YLnphT7IPA strain, so the seeding concentration was increased to the initial O.D. twofold when cultured with crude glycerol in a fermenter. This allowed for the growth rate of the strain to be alleviated in the first 24 h (Fig. 7). Consumption of crude glycerol by the engineered strain produced 1.60 g/L isopropanol after 48 h of fermenter cultivation.

As shown in Fig. 4, in the fermenter, the yield of isopropanol in crude glycerol medium reached 82.52% of that in pure glycerol medium. Compared with the pure glycerol medium, the decrease in isopropanol production may be due to the increase in the concentration of impurities caused by a large amount of crude glycerol input during culture in the fermenter, which affects the metabolism of the YLnphT7IPA strain to a certain extent. Therefore, the 17.48% reduction in production is acceptable and thus this is a highly successful strategy.

## Conclusion

This study is the first to synthesize of isopropanol in *Y. lipolytica*. The most suitable isopropanol synthesis pathway increased the isopropanol yield by integrating heterologous *nphT7*, *atoDA*, *adc*, and *adh*. The maximum titer of isopropanol synthesis by *Y. lipolytica* reported to date was obtained after cultivation in a fermenter using the optimized medium developed by response surface methodology. In conclusion, this is the first study to biosynthesize isopropanol using crude glycerol as a carbon source, and a similar isopropanol yield compared to that using pure glycerol as a carbon source was obtained. This study demonstrates the ability of engineered *Y. lipolytica* to effectively utilize industrial crude glycerol for biofuel production.

## Materials and methods

### Strains and plasmids

The plasmids, primers and strains used are shown in Additional file 1: Table S1. The genomes of *Clostridium acetobutylicum* ATCC 842 and *E. coli* K12 were used as the PCR templates. Gene segments of *adc* and *thl* from *C. acetobutylicum* ATCC 842 genomic DNA and *atoDA* from *E. coli* K12 genomic DNA were amplified by PCR. Genomic DNA Purification Kit (Promega, Wisconsin, U.S.A.) was used for all genomic DNA extractions. The gene encoding secondary alcohol dehydrogenase (*adh*) from *Clostridium beijerinckii* NRRL B593 and the acetoacetyl-CoA synthase (*nphT7*) gene from *Streptomyces* sp. (strain CL190) were synthesized by Cosmogenetech (Cosmogenetech, Seoul, Korea) with codon optimization for use in yeast. All synthesized gene sequences are provided in Additional file 1: Table S2. The following steps were taken to produce recombinant plasmids. The *atoDA* and *adh* genes were amplified with the primer pairs, SpeI_atoDA_F/ClaI_atoDA_R and Sac1_adh_F/Pac1_adh_R, respectively, and inserted into the corresponding restriction site in the pSPG1 vector, resulting in the plasmid pSPG1-atoDA-adh. Using pSPG1-atoDA-adh as the template, the *atoDA-adh*-linked genes were amplified with the primer pairs, PmeI_atoDAadh_F and BamHI_atoDAadh_R and inserted into the corresponding restriction site in the pYLEX1 expression vector containing the strong hybrid promoter (hp4d) and XPR2 terminator and followed by a leucine selection marker gene (LEU2) resulting in the plasmid pYLEX1-atoDA-adh. The *adc* gene was amplified with the primer pairs, Xcm1_adc_F and Kpn1_adc_F inserted in the corresponding restriction site into pYLEX1-atoDA-adh, resulting in the plasmid pYLEX1IPA. T4 DNA ligase (Enzynomics, Daejeon, Korea) was used to ligate genes into their respective restriction enzyme sites in the plasmid. The plasmid pYLEX1THLIPA was constructed by amplifying the *thl* gene with primer pairs for Gibson assembly, Gibson_thl_F/Gibson_thl_R inserted in pYLEX1IPA using a Gibson Assembly Cloning Kit (NEB Gibson Assembly Master Mix, #E2011) [27]. The sample was incubated for 1 h at 50 °C using the Gibson cloning master mix. The plasmid pYLEX1NPHT7IPA was created by amplifying the gene *nphT7* with the primer pair Gibson_nphT7_F/Gibson_nphT7_R inserted into the plasmid pYLEX1IPA using the Gibson Assembly Cloning Kit. Plasmids were transformed into *E. coli* DH5α, and heat shock was used to perform the transformation. Then, the cells were plated on Luria–Bertani (LB) agar with ampicillin added for positive transformant selection. Recombinant *E. coli* cells were selected and cultured in LB media containing ampicillin for overnight. After PCR detection, the cloned vector was purified and confirmed by sequencing (Cosmogenetech, Seoul, Korea). Extracted plasmids from *E. coli* were then transformed into *Y. lipolytica* Po1g. After digestion with *NotI*, the plasmid pYLEX1NPHT7IPA and pYLEX1THLIPA were linearized to insert genes into the *Y. lipolytica* genome. A YLEX Yeast Expression Kit (Yeastern Biotech Co., Ltd.) was used for *Y. lipolytica* Po1g transformation. Then, the cells were plated on SD-Leu agar for transformant selection.

### Cultivation conditions

For molecular genetic procedures, LB medium (10 g/L NaCl, 10 g/L tryptone, and 5 g/L yeast extract) was used. LB medium was autoclaved at 120 ℃ for 15 min. After cooled to room temperature, ampicillin (50 μg/mL) was added to the LB medium to obtain LA medium and used for recombinant *E. coli* cell selection. The *Y. lipolytica* transformant was selected and cultured at 30 °C and 200 rpm in minimal SD-Leu medium (6.7 g/L yeast nitrogen base without amino acids, 0.69 g/L leucine dropout amino acids, and 20 g/L glucose). The recombinant *Y. lipolytica* cell was selected and cultured in YPD medium (10 g/L yeast extract, 20 g/L peptone, and 20 g/L glucose). Engineered *Y. lipolytica* strains were cultivated in YPG medium (10 g/L yeast extract, 20 g/L peptone, and 20 g/L glycerol) for preculture and YPG 80 medium (10 g/L yeast extract, 20 g/L peptone, and 80 g/L glycerol) for isopropanol production. The YP-crude glycerol medium (crude glycerol as a carbon source) contains the following components. First, crude glycerol containing glycerol 78% w/w, soap 2.4% w/w, methanol 1.28% w/w, water 2.48% w/w and NaOH 0.12% w/w from the biodiesel industry was prepared according to previous reports [28, 29]. Then according to the required concentration of glycerol in the optimal medium, the 70 g/L crude glycerol was added to the optimal YP medium (20 g/L yeast extract and 35 g/L peptone). Then, according to the required concentration of glycerol in the optimal medium, 70 g/L crude glycerol was added to the optimal YP medium (20 g/L yeast extract and 35 g/L peptone). After 48 h of preculture in YPG medium, the cells were centrifuged at 8000 rpm for 2 min, and the supernatants were discarded. The cells were washed twice with sterile water and then centrifuged at 8000 rpm for 2 min again. After the washing steps, the pure glycerol used in preculture was removed and then the cells were transferred into 50 ml YP-Crude glycerol medium in 250 ml flask and cultured at 30 °C and 200 rpm for 96 h. The samples were collected every 12 h during the flask culture, and the samples were stored in the sterilized centrifuge tube. All operations were completed in the clean bench.

### Medium optimization by response surface methodology (RSM)

Box–Behnken design (BBD) was adopted as the experimental design model to optimize the YPG medium to improve the yield of isopropanol. The experimental results were statistically analyzed using ANOVA (Table 2). The experimental design was determined by Design-Expert 7.0.0. Central composite design (CCD) was adopted as the experimental design model to optimize YPD medium for isopropanol production in glucose. ANOVA was used to statistically analyze the experimental results. Additional file 1: Table S3 shows the experimental design. The culture was performed in 250 mL shake flasks with 50 mL culture medium and incubated at 30 °C and 200 rpm for 72 h. Samples were taken every 24 h.

### Fed-batch fermentation

After three days of streaking on SD-Leu plates, the YLnphT7IPA strain was transferred to preculture YPG medium (10 g/L yeast extract, 20 g/L peptone, and 20 g/L glycerol). Preculture was carried out for 48 h at 200 rpm at 30 °C before being transferred to 50 ml of YPG 80 medium for the second seed culture. The second seed broth was inoculated into a 5 L fermenter containing 2 L optimal YPG medium (20 g/L yeast extract, 35 g/L peptone, and 70 g/L glycerol). Fermentation was performed at 30 °C and 200–700 rpm for 60 h. Then, 3 M H_2_SO_4_ and 3 M KOH were used to adjust the pH to 5.89. For the batch culture of YP-crude glycerol medium, the same seed was inoculated into a 5 L fermenter containing 2 L of optimal YP-crude glycerol medium (20 g/L yeast extract, 35 g/L peptone, and 70 g/L crude glycerol). The same steps were used for batch culture of YPD medium, and the YLnphT7IPA strain was precultured in YPD medium (10 g/L yeast extract, 20 g/L peptone, and 20 g/L glucose). After 48 h at 200 rpm at 30 °C, the precultured strain was transferred to 50 ml of YPD 80 medium for the 48 h second seed culture. Then, this seed broth was inoculated into a fermenter with 2 L of optimal YPD medium (17.5 g/L yeast extract, 40 g/L peptone, and 93 g/L glucose). Fermentation was performed at 30 °C and 200–700 rpm for 48 h. The dissolved oxygen (DO) was maintained at 20%. The pH was adjusted to 5.89 using 3 M H_2_SO_4_ and 3 M KOH. Samples were taken every 6 h.

### Analytical methods

A UV–vis spectrophotometer was used to measure the cell growth of *Y. lipolytica* at OD_600_ (Mecasys, Seoul, Korea). After the cultured samples were centrifuged at 13,000 rpm for 3 min, the supernatant was taken for machine analysis. Isopropanol was analyzed using a high-performance gas chromatograph (GC) (model GC7890; Agilent, Santa Clara, CA) equipped with a flame ionization detector (FID) and a DB-WAXetr column (Agilent, 30 m, 0.32 mm, 0.25 µm). The GC oven temperature was set at 40 °C for 1 min, then gradually increased to 100 °C using a 4 °C/min gradient and held for 1 min. The detector temperature was kept constant at 250 °C. Nitrogen was used as a carrier gas. Glycerol was analyzed using a high-performance liquid chromatography (HPLC) system (binary HPLC pump Model 1528, autosampler Model 2707, Refractive Index Detector, Waters, MA, U.S.A.) using an HPLC column (HPLC column Hi-Plex H, 8 µm, 7,7 × 300 mm). 0.0085 M sulfuric acid was used as the mobile phase with a flow rate of 0.6 ml/min. The oven temperature was set to 75 °C, and the RID detector temperature was set to 50 °C.

## Supplementary Information

**Additional file 1: Table S1.** Heterologous genes, strains, plasmids, and primers used in this study. **Table. S2.** Synthesized gene sequences. **Table S3.** Level of factors chosen for the central composite design response surface methodology and ANOVA analysis of the model obtained for optimal medium. **Table S4**. Isopropanol yield results optimal YPD medium for CCD-RSM. **Figure S1.** YLnphT7IPA strain was cultured in 5 L fermenter using glucose as carbon source.

## Data Availability

All data generated or analyzed during this study are included in this published article [and its Additional files].

## Abbreviations

*thl*: Acetyl-CoA acetyltransferase
*nphT7*: Acetoacetyl-CoA synthase
*atoDA*: Acetoacetyl-CoA transferase
*adc*: Acetoacetate decarboxylase
*adh*: Secondary alcohol dehydrogenase
*Y. ** lipolytica*: *Yarrowia* *lipolytica*
*E. coli*: *Escherichia* *coli*
GRAS: Generally Recognized As Safe
RSM: Response Surface Methodology
CCD: Central composite design
BBD: Box–Behnken design
FID: Flame ionization detector
HPLC: High-performance liquid chromatography
GC: Gas chromatograph
RID: Refractive Index Detector
YP: Yest peptone
YPD: Yest peptone glucose
YPG: Yest peptone glycerol
LB: Luria broth
OD: Optical density
