# Supplementary material for: Isopropanol biosynthesis from crude glycerol using fatty acid precursors via engineered oleaginous yeast Yarrowia lipolytica
Source: Microb Cell Fact. 2022 Aug 19;21:168. doi: 10.1186/s12934-022-01890-6 (PMC9392242; doi:10.1186/s12934-022-01890-6)
Supplement: Supplementary file 1 — Additional file 1: Table S1. Heterologous genes, strains, plasmids, and primers used in this study. Table. S2. Synthesized gene sequences. Table S3. Level of factors chosen for the central composite design response surface methodology and ANOVA analysis of the model obtained for optimal medium. Table S4. Isopropanol yield results optimal YPD medium for CCD-RSM. Figure S1. YLnphT7IPA strain was cultured in 5 L fermenter using glucose as carbon source. [file 12934_2022_1890_MOESM1_ESM.docx]

**Additional file 1**

**Isopropanol Biosynthesis from Crude Glycerol using Fatty Acid Precursors via Engineered Oleaginous Yeast *Yarrowia lipolytica*.**

Xiaoyu Shi ^1^, Hyeon Min Park ^1^, Minhye Kim^1^, Myeong-Eun Lee^1^, Wu-Young Jeong^1^, Joonhee Chang^1^, Byeong-Hyeon Cho^1^ and Sung Ok Han^1*^

1 Department of Biotechnology, Korea University, Seoul 02841, Republic of Korea

* Corresponding author

**Table S1.** **Heterologous genes, strains, plasmids, and primers used in this study.**

| **Genes** | **Protein name** | | **Organism** |
| --- | --- | --- | --- |
| thl | acetyl-coenzyme A acetyltransferase | | *Clostridium acetobutylicum* ATCC 842 |
| nphT7 | Acetoacetyl CoA synthase | | *Streptomyces sp.* (strain CL190) |
| atoDA | Acetate CoA-transferase subunit alpha/beta | | *Escherichia coli* (strain K12) |
| adc | acetoacetyl-CoA transferase | | *Clostridium acetobutylicum* ATCC 842 |
| sadh | NADP-dependent isopropanol dehydrogenase | | *Clostridium beijerinckii* NRRL B593 |
| **Strain** | | **Relevant characteristics** | |
| Yarrowia lipolytica Po1g | | Wild type | |
| YLIPA | | *atoDA-adc-adh* integrated in *Yarrowia lipolytica* genome | |
| YLthlIPA | | *thl-atoDA-adc-adh* integrated in *Yarrowia lipolytica* genome | |
| YLnphT7IPA | | *nphT7-atoDA-adc-adh* integrated in *Yarrowia lipolytica* genome | |
| **Plasmid** | | **Relevant characteristics** | |
| pSPG1-atoDA-adh | | P_TEF1_::atoDA,adh | |
| pYLEX1-atoDA-adh | | P_hp4d_::atoDA,adh | |
| pYLEX1IPA | | P_hp4d_::atoDA,adc,adh | |
| pYLEX1THLIPA | | P_hp4d_::thl,atoDA,adc,adh | |
| pYLEX1NPHT7IPA | | P_hp4d_::nphT7,atoDA,adc,adh | |
| **Primers** | |  | |
| SpeI_atoDA_F | | GCGCACTAGTATGAAAACAAAATTGATGACAT | |
| ClaI_atoDA_R | | TTTAATCGATTCATAAATCACCCCG | |
| Sac1_adh_F | | GCAGAGCTCATGAAAGGATTTGCTATGCT | |
| Pac1_adh_R | | CTTAATTAATTACAGGATAACCACTGCCTT | |
| PmeI_atoDAadh_F | | GCGCCACGTGATGAAAACAAAATTGATGACAT | |
| BamHI_atoDAadh_R | | TAAGGATCCTTACAGGATAACCACTGCCTT | |
| Xcm1_adc_F | | ATCCAGTCCGACTCTGGATGTTAAAGGATGAAGTAATTAAACAAATTAGCAC | |
| Kpn1_adc_R | | CCGGTACCTTACTTAAGATAATCATATATAACTTCAGCTCTAGGC | |
| Gibson_thl_F | | TACAACCACACACATCCACAATGAAAGAAGTTGTAATAG | |
| Gibson_thl_R | | CAATTTTGTTTTCATCACCTAGCACTTTTCTAGCAATA | |
| Gibson_nphT7_F | | TACAACCACACACATCCACAATGACGGATGTTCGATTCC | |
| Gibson_nphT7_R | | CAATTTTGTTTTCATCACCTACCACTCGATAAGTGCG | |

**Table S2. Synthesized gene sequences**

| Gene | Sequence |
| --- | --- |
| Acetoacetyl-CoA synthase | ATGACGGATGTTCGATTCCGAATTATTGGTACCGGTGCCTATGTTCCCGAACGAATCGTCTCTAATGACGAAGTGGGAGCACCTGCTGGCGTGGATGACGATTGGATTACAAGAAAGACAGGAATTAGACAACGGCGGTGGGCAGCTGACGATCAGGCAACGTCTGATCTTGCAACTGCCGCCGGTCGAGCAGCACTGAAGGCTGCTGGCATTACCCCTGAACAGCTTACTGTGATCGCAGTGGCCACTTCCACACCCGACAGACCCCAACCTCCTACGGCAGCTTACGTTCAACATCACCTCGGTGCTACTGGAACTGCCGCATTTGATGTCAACGCTGTGTGTTCCGGCACGGTCTTCGCCCTTTCCTCGGTGGCAGGTACGCTCGTCTATCGTGGAGGCTACGCCCTCGTCATTGGTGCCGACCTTTATTCGCGGATCCTGAACCCCGCAGATCGGAAGACCGTTGTGCTCTTTGGAGATGGTGCCGGTGCAATGGTCCTCGGCCCTACATCCACTGGAACTGGTCCTATTGTCCGTCGGGTCGCTCTCCACACCTTCGGAGGACTCACAGATCTGATTAGAGTGCCCGCCGGAGGTTCCCGTCAGCCTCTCGATACAGATGGACTCGATGCAGGTCTGCAGTATTTTGCAATGGATGGACGGGAAGTGCGACGGTTCGTTACTGAACATCTTCCTCAACTCATTAAAGGATTTCTGCATGAAGCAGGCGTTGATGCTGCCGACATTAGCCACTTCGTCCCCCATCAGGCAAATGGAGTTATGCTCGACGAGGTTTTTGGAGAGCTCCATCTTCCCAGAGCCACTATGCACCGGACCGTCGAGACTTATGGAAACACTGGAGCCGCATCGATCCCCATTACAATGGACGCTGCCGTTCGAGCTGGTTCCTTTCGTCCCGGCGAGCTGGTTCTTCTGGCTGGATTTGGTGGTGGCATGGCTGCCAGCTTCGCACTTATCGAGTGGTAG |
| Secondary alcohol dehydrogenase | ATGAAAGGATTTGCTATGCTAGGCATAAACAAGTTAGGTTGGATCGAAAAGGAACGTCCGGTAGCAGGAAGTTATGACGCAATTGTTAGACCATTGGCAGTGAGCCCATGTACATCAGACATACACACTGTGTTTGAGGGTGCACTGGGCGATAGAAAAAATATGATCTTAGGCCATGAAGCTGTCGGAGAAGTCGTGGAGGTTGGGAGTGAAGTCAAGGATTTTAAGCCAGGAGATAGAGTAATTGTTCCTTGTACTACCCCAGACTGGCGTAGTTTGGAGGTCCAAGCAGGGTTTCAACAACACTCCAATGGCATGTTGGCGGGGTGGAAGTTCTCTAATTTCAAAGATGGTGTTTTTGGTGAGTATTTCCACGTCAATGATGCGGATATGAATTTGGCGATCCTTCCTAAAGACATGCCGCTTGAAAATGCTGTTATGATTACTGATATGATGACCACGGGATTTCATGGGGCAGAACTTGCTGATATCCAAATGGGCTCATCTGTTGTAGTGATTGGTATAGGAGCTGTTGGCCTTATGGGTATCGCCGGGGCGAAGTTAAGGGGCGCTGGCCGTATAATAGGGGTAGGCTCTAGGCCCATTTGCGTTGAGGCTGCTAAGTTTTATGGAGCAACTGACATTCTTAATTACAAGAATGGTCATATAGTAGACCAAGTTATGAAGCTTACCAACGGTAAGGGTGTAGATAGGGTGATCATGGCGGGTGGAGGTAGCGAAACTTTAAGTCAGGCTGTATCTATGGTCAAGCCAGGAGGCATTATTAGCAACATAAACTATCATGGCTCCGGCGACGCGTTGTTAATTCCAAGAGTCGAGTGGGGTTGCGGGATGGCACACAAAACAATCAAAGGTGGATTGTGCCCCGGGGGCCGTCTACGTGCTGAGATGTTGCGTGATATGGTGGTCTATAATCGTGTCGATTTGAGTAAGTTAGTCACCCATGTGTACCATGGATTTGACCATATAGAAGAGGCACTGTTACTAATGAAGGACAAACCGAAAGATTTGATAAAGGCAGTGGTTATCCTGTAA |

**Table S3.** **Level of factors chosen for the central composite design response surface methodology and ANOVA analysis of the model obtained for optimal medium**

| Factor | Name | | Low actual | | High Actual | | Low Coded | | High Coded | | Mean | | Std. Dev |
| --- | --- | --- | --- | --- | --- | --- | --- | --- | --- | --- | --- | --- | --- |
| A | Yeast extract (g/L) | | 10.00 | | 25.00 | | -1.000 | | 1.000 | | 17.500 | | 6.706 |
| B | Peptone (g/L) | | 20.00 | | 50.00 | | -1.000 | | 1.000 | | 34.917 | | 13.424 |
| C | Glucose (g/L) | | 57.50 | | 132.50 | | -1.000 | | 1.000 | | 95.000 | | 33.541 |
| D | pH | | 5.00 | | 8.00 | | -1.000 | | 1.000 | | 6.5000 | | 1.342 |
| Source | | Sum of squares | | Df | | Mean square | | F value | | P-value Prob>F | |  | |
| Model | | 14.16 | | 14 | | 1.01 | | 2.71 | | 0.0324 | | *significant* | |
| A-Yeast extract | | 0.099 | | 1 | | 0.099 | | 0.27 | | 0.6141 | |  | |
| B-Peptone | | 0.46 | | 1 | | 0.46 | | 1.25 | | 0.2818 | |  | |
| C-Glucose | | 0.12 | | 1 | | 0.12 | | 0.33 | | 0.5739 | |  | |
| D-Ph | | 0.38 | | 1 | | 0.38 | | 1.03 | | 0.3259 | |  | |
| AB | | 0.19 | | 1 | | 0.19 | | 0.51 | | 0.4875 | |  | |
| AC | | 6171E-003 | | 1 | | 6.171E-003 | | 0.017 | | 0.8994 | |  | |
| AD | | 0.11 | | 1 | | 0.11 | | 0.28 | | 0.6033 | |  | |
| BC | | 0.10 | | 1 | | 0.10 | | 0.27 | | 0.6121 | |  | |
| BD | | 0.068 | | 1 | | 0.068 | | 0.18 | | 0.6763 | |  | |
| CD | | 1.441E-003 | | 1 | | 1.441E-003 | | 3.863E-003 | | 0.9513 | |  | |
| A^2^ | | 5.80 | | 1 | | 5.80 | | 15.56 | | 0.0013 | |  | |
| B^2^ | | 1.96 | | 1 | | 1.96 | | 5.25 | | 0.0368 | |  | |
| C^2^ | | 7.87 | | 1 | | 7.87 | | 21.12 | | 0.0004 | |  | |
| D^2^ | | 0.88 | | 1 | | 0.88 | | 2.36 | | 0.1451 | |  | |
| Lack of Fit | | 4.49 | | 10 | | 0.45 | | 2.03 | | 0.2251 | | *not significant* | |
| R^2^ | |  | |  | |  | | 0.7168 | |  | |  | |
| Adj R^2^ | |  | |  | |  | | 0.4525 | |  | |  | |
| Pred R^2^ | |  | |  | |  | | -0.3899 | |  | |  | |
| *Adeq. Precision* | |  | |  | |  | | 5.2960 | |  | |  | |

**Table S4.** **Isopropanol yield results optimal YPD medium for CCD-RSM**

| Run | Yeast extract (g/L) | Peptone (g/L) | Glucose (g/L) | pH | Isopropanol (g/L) |
| --- | --- | --- | --- | --- | --- |
| 1 | 17.50 | 35.00 | 95.00 | 6.50 | 2.01 |
| 2 | 17.50 | 65.00 | 95.00 | 6.50 | 2.011 |
| 3 | 17.50 | 35.00 | 95.00 | 6.50 | 2.15 |
| 4 | 10.00 | 20.00 | 57.50 | 5.00 | 0.187 |
| 5 | 17.50 | 35.00 | 95.00 | 3.50 | 2.455 |
| 6 | 32.50 | 32.50 | 95.00 | 6.50 | 0.72 |
| 7 | 10.00 | 50.00 | 132.50 | 5.00 | 0.159 |
| 8 | 17.50 | 35.00 | 170.00 | 6.50 | 0.101 |
| 9 | 25.00 | 20.00 | 57.50 | 5.00 | 0.244 |
| 10 | 2.50 | 35.00 | 95.00 | 6.50 | 0.1109 |
| 11 | 17.50 | 35.00 | 95.00 | 9.50 | 0.61931 |
| 12 | 25.00 | 50.00 | 132.50 | 8.00 | 0.297 |
| 13 | 10.00 | 50.00 | 57.00 | 8.00 | 0.205 |
| 14 | 17.50 | 35.00 | 95.00 | 6.50 | 1.00 |
| 15 | 10.00 | 20.00 | 132.50 | 8.00 | 0.0692 |
| 16 | 25.00 | 20.00 | 132.50 | 5.00 | 0.065 |
| 17 | 10.00 | 50.00 | 57.50 | 5.00 | 0.68 |
| 18 | 17.50 | 5.00 | 95.00 | 6.50 | 0.3545 |
| 19 | 25.00 | 20.00 | 132.50 | 8.00 | 0.04338 |
| 20 | 17.50 | 35.00 | 95.00 | 6.50 | 1.98 |
| 21 | 10.00 | 20.00 | 132.50 | 5.00 | 0.0561 |
| 22 | 10.00 | 50.00 | 132.50 | 8.00 | 0.29 |
| 23 | 25.00 | 50.00 | 57.50 | 8.00 | 0.1353 |
| 24 | 17.50 | 35.00 | 95.00 | 6.50 | 1.79 |
| 25 | 17.50 | 35.00 | 20.00 | 6.50 | 0.12 |
| 26 | 25.00 | 50.00 | 132.50 | 5.00 | 0.179 |
| 27 | 25.00 | 50.00 | 57.50 | 5.00 | 0.113 |
| 28 | 17.50 | 35.00 | 95.00 | 6.50 | 2.35294 |
| 29 | 25.00 | 20.00 | 57.50 | 8.00 | 1.09 |
| 30 | 10.00 | 20.00 | 57.50 | 8.00 | 0.186 |


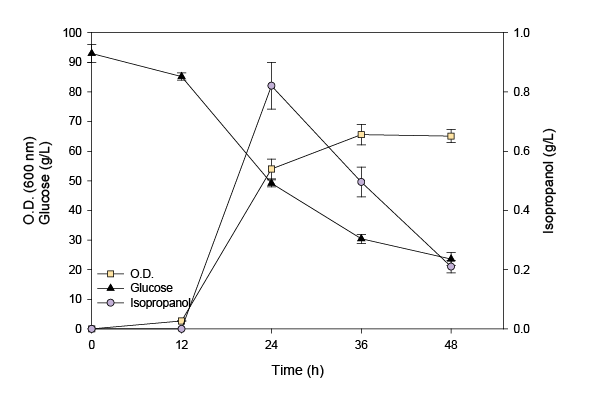


**Figure S1.**

**YLnphT7IPA strain was cultured in 5 L fermenter using glucose as carbon source.** Line in yellow, purple, and black represented growth curves, isopropanol production and glucose consumption, respectively. The quantification was conducted in triplicate.
